# The orphaning experience: descriptions from Ugandan youth who have lost parents to HIV/AIDS

**DOI:** 10.1186/1753-2000-4-6

**Published:** 2010-02-07

**Authors:** Sheila Harms, Susan Jack, Joshua Ssebunnya, Ruth Kizza

**Affiliations:** 1 Department of Psychiatry and Behavioural Neurosciences, McMaster University, 3G - Child and Youth Mental Health Unit, 1200 Main St. West, Hamilton, Ontario, L8N 3Z5, Canada; 2 School of Nursing, McMaster University, 1200 Main St. West, Hamilton, Ontario, L8N 3Z5, Canada; 3 Department of Mental Health and Community Psychology, Makerere University, P.O. Box 7062, Kampala, Uganda; 4 Department of Psychiatry, Makerere University, P.O. Box 7062, Kampala, Uganda

## Abstract

The HIV/AIDS epidemic has continued to pose significant challenges to countries in Sub-Saharan Africa. Millions of African children and youth have lost parents to HIV/AIDS leaving a generation of orphans to be cared for within extended family systems and communities. The experiences of youth who have lost parents to the HIV/AIDS epidemic provide an important ingress into this complex, evolving, multi-dimensional phenomenon. A fundamental qualitative descriptive study was conducted to develop a culturally relevant and comprehensive description of the experiences of orphanhood from the perspectives of Ugandan youth. A purposeful sample of 13 youth who had lost one or both parents to HIV/AIDS and who were affiliated with a non-governmental organization providing support to orphans were interviewed. Youth orphaned by HIV/AIDS described the experience of orphanhood beginning with parental illness, not death. Several losses were associated with the death of a parent including lost social capitol, educational opportunities and monetary assets. Unique findings revealed that youth experienced culturally specific stigma and conflict which was distinctly related to their HIV/AIDS orphan status. Exploitation within extended cultural family systems was also reported. Results from this study suggest that there is a pressing need to identify and provide culturally appropriate services for these Ugandan youth prior to and after the loss of a parent(s).

## Background

In Sub-Saharan Africa, a significant and catastrophic long-term outcome of the HIV/AIDS crisis has been the emergence of a large population of children and youth who have lost one or both parents to this disease. In this region, approximately 12 to 14 million children have lost at least one parent to HIV/AIDS, resulting in the highest prevalence of this category of orphans worldwide [1]. Using conservative estimates, UNICEF has postulated that the orphan population may reach 18 million by the year 2010 [2]. Over the past 10 years, there has been a significant increase in research exploring this complex, evolving, multi-dimensional phenomenon.

The current body of literature related to the orphan experience of African youth has produced important quantitative research findings that have resulted in taxonomies attempting to capture the current HIV/AIDS orphan phenomenon. Epidemiological studies have been conducted to characterize living and care giving arrangements for orphans. Additional studies have reported on the academic, physical and mental health outcomes of HIV/AIDS orphans. However, the individual experience of orphanhood as expressed through the personal perspectives of orphaned youth has been comparatively neglected, resulting in a current body of literature that is somewhat unbalanced. This study has attempted to fill this gap by providing narratives from orphaned youth who have articulated personal stories as they pertain to their early experiences of orphanhood secondary to HIV/AIDS.

While the current qualitative study is unique with its primary objective of exploring youth narratives related to the orphaning experience, a number of qualitative studies exploring the various facets of the HIV/AIDS orphan phenomenon in Africa have reported secondary data which have addressed the orphan story from the youth perspective. Although there are some reports of hope and resilience, recent studies have provided a foray into life experiences marked by significant difficulties and unsuspected life changes, emotional pain, fears about the future, stigma and losses. In a study by Wood and colleagues [3], Zimbabwean orphaned youth described the demands associated with parental illness as a significant challenge. Specifically, youth reported growing up and needing to behave like adults because of the emotional and care-giving demands placed on them. Youth also spoke about the secrecy which marked traditional approaches to death. Adolescents were commonly unaware of the nature of their parents' illnesses and articulated the need to be aware of the cause of illness and death. The importance of silence as a virtuous coping strategy was emotionally very difficult, particularly for older siblings who assumed responsibility for their younger siblings. However, many youth found comfort and a sense of distraction if they had the ability to attend school.

Satzinger, Kipp and Rubaale [4] conducted a qualitative study in Uganda examining HIV/AIDS orphans' personal health concerns. Ongoing fears about a number of potential threats to their health or well-being, including the fear of being robbed or exploited within their own communities were reported. Not being able to produce enough food to adequately feed themselves and their siblings resulted in feelings of constant hunger. Cluver and Gardner [5] studied risk and protective factors for psychological well-being of children orphaned by HIV/AIDS in South Africa. One notable finding was the tension articulated by youth when they were compared to biological children and referred to as orphans within their new family situations. Poverty was also a common theme which affected the capacity for basic care giving, access to health services and education. Similar findings were reported in a study conducted in Uganda by Harms and colleagues [6] where perceptions of mental health as it related to their orphan status were explored.

The experiences of youth orphaned by HIV/AIDS also include examining caregiving arrangements for this population as an important foray into the familial and cultural context for the orphan crisis in Sub-Saharan Africa. A large qualitative study examining social dynamics of orphan care in various Ethiopian families [7] challenged two existing theoretical interpretations related to orphan care. The first theory assumed that the current HIV/AIDS crisis has stretched the traditional African extended family system beyond its capacity to provide adequate care. The second theory suggested that resiliencies and strengths exist within traditional family systems if they are adequately supported. Their findings suggested that the extended family care arrangements required to support orphans is contextually driven and can be understood along a continuum of different family typologies ranging from disrupted to capable families.

Oleke and colleagues [8] conducted a large ethnographic study in Northern Uganda to examine the cultural factors related to orphan care. Specifically, they found that the patrilineal cultural practices which would historically ensure financial stability and safety for orphans have changed over time. Their findings suggested that orphaned children were being cared for by elderly female care-givers, increasing the vulnerability of both orphans and their elderly caregivers. Nyambedha and colleagues [9] also concluded that high numbers of orphans had overwhelmed the patrilineal patterns of care in Western Kenya such that matrilineal clan ties or strangers were looking after 28% of orphans in their study. This shift was described as a notable deviation from the typical cultural norms. Negative attitudes towards orphans, poverty and old age of care-givers were additional challenges.

One longitudinal study [10] collected orphan incidence and prevalence data from several African countries to better understand their living arrangements. Relatively few child-headed households were identified. However, depending on cultures and clan arrangements, most orphaned children and youth were cared for by close or distant relatives, with grandmothers being an important group of caregivers in South Africa. A recent systematic review by Kuo and Operario [11]examining studies on caregivers for HIV/AIDS orphans revealed that elderly females within the extended family system have assumed the responsibility of care, despite potential risks to their psychological and physical health. These caregiving duties are associated with financial stressors and potential lost social support in weakening family systems. Barriers for others to assume care giving roles are primarily financial in nature. A number of studies have concluded that African homes where HIV/AIDS orphan care responsibilities have been adopted experience significant financial stress [12-14] such that large scale financial contributions are required to support orphans and other vulnerable children to meet essential needs.

While the current HIV/AIDS orphan crisis has challenged existing family systems, data have emerged suggesting that HIV/AIDS orphans are affected at an individual level. Specifically, a number of negative health effects for orphans have been reported. In a 2007 study conducted in Botswana [15], HIV/AIDS orphans as compared to non-orphans were more likely to experience poor physical health outcomes and higher mortality rates, regardless of their own HIV status. Differing results were obtained in a Kenyan cross-sectional demographic health survey which included 2756 children ages 0 to 4 years and 4172 children and youth ages 6 to 14 years. Odds ratios indicated that orphans were less likely to be stunted, equally as likely to be underweight and more likely to be wasted compared to children of HIV-negative parents. Comparisons between orphans and non-orphaned children on access to healthcare were not presented [16]. At the individual and family level, biological illnesses, non-compliance with treatment regimes, poverty and care-giver illness play a role, as do the broader organizational and social factors such as reduced access to health care services and fragmented health care infrastructures.

In addition to negative health outcomes, recent findings have suggested that there are specific psychological effects associated with being an HIV/AIDS orphan. Cluver and Gardner [5] examined psychological risk and protective factors in South African HIV/AIDS orphans using descriptive methodology. In their study, 60 HIV/AIDS orphans (defined as a child or youth under the age of 18 who had lost one or both parents to HIV/AIDS), 42 orphan caregivers and 20 care professionals were interviewed. Children were recruited from schools, street agencies and welfare services. Key findings from this study suggested that being cared for by a capable, competent loving caregiver, being involved with the extended family, attending school and having access to social services were described as protective factors. Factors associated with poor psychological well-being included bereavement, abuse, stigmatization and discrimination, poverty, inequities within the home and a lack of care providers.

Although the experience of being an HIV/AIDS orphan has not been associated with any one specific psychological profile, a general trend has emerged suggesting that HIV/AIDS orphans experience a higher level of, or are more vulnerable to: psychological distress including internalizing symptoms such as depression and anxiety; social difficulties; and behavioural disturbances compared to their non-orphaned peers [17-24]. Other possible indicators of orphan well-being have also been examined. Nyamukapa et al. [23] developed a theoretical framework to explain causal factors for psychosocial distress and consequences for Zimbabwean orphaned youth using in a large scale cross sectional study using data from a 2004 national survey. Findings from this study suggested that psychosocial distress associated with orphanhood led to early sexual experiences. Psychological distress was a mediating factor for poverty status as well as not being in school although conclusions in this regard were less convincing. Other studies have found that orphan status is associated with an increase in early sexual behaviours as compared to non-orphans [25].

In summary, literature focused on outcomes related to the HIV/AIDS orphan phenomenon in Sub-Saharan Africa indicates that care-giving arrangements are changing over time, perhaps reflecting an erosion of culturally-traditional supports. While there are some exceptions, physical, psychological and behavioural outcomes for HIV/AIDS orphans are generally less robust compared to their non-orphan counterparts. However, many of the studies measuring outcomes of HIV/AIDS orphans are characterized by specific methodological weaknesses which necessitates caution when interpreting and comparing findings. Methodological challenges in this field of study have included the lack of standard definitions for the term "orphan" as well as difficulties confirming orphanhood status secondary to parental HIV/AIDS related death. Additional challenges have included a lack of control groups and the use of non-standardized, non-culturally validated instruments in studies measuring different health outcomes. Additionally many of the health outcome studies have been conducted using cross-sectional designs which increases the potential for erroneous conclusions about changes in outcomes over time. Finally, the dynamic multi-faceted nature of the HIV/AIDS orphanhood phenomena in Africa requires the use of a variety of research methods, both quantitative and qualitative, to ensure that the phenomenon is comprehensively and holistically described and understood.

Qualitative research offers the opportunity for knowledge construction that is contextually and culturally situated which is critical to understanding a complex phenomenon such as the current African HIV/AIDS orphan crisis [26]. In a recent qualitative descriptive study by Harms and colleagues [6] with 13 purposefully selected Ugandan youth orphaned by HIV/AIDS who were being supported by non-governmental agencies (NGOs), a central question about youths' perceptions and definitions of mental health as it related to their orphan status was explored. The goal of the study was to gather data that would inform the construction of a culturally validated tool to measure mental health outcomes of relevance to this population [27]. Results indicated that youth struggled with ongoing emotional and psychological difficulties following the deaths of their parents, which was attributed to hardships such as poverty, loss of educational opportunities, as well as exploitation and conflict in their surrogate homes. Culturally specific terminology was identified which served as descriptors for some of the negative psychological phenomena. Conceptions of psychologically resilient orphans were linked to social conduct and abilities to respect cultural norms.

While our central focus was to conduct an in-depth exploration of youths' perceptions of their mental health and how their orphanhood status influenced changes in mental health, the study participants shared extensive details about the process and experience of becoming an orphan. The objective of the current article is to describe the experiences of entering into orphanhood from the perspectives of Ugandan youth orphaned by HIV/AIDS. While other studies have reported similar secondary data, the importance of this study describing the orphaning experience from the direct viewpoint of the youth provides important foundational information for the deeper exploration of this issue and offers a new perspective to view this phenomenon under study [27]. With this new understanding, clinicians or individuals working with HIV/AIDS orphans through NGOs might utilize this information to provide increasingly empathic care and anticipatory guidance to the youth they work with in their respective organizations.

## Methods

### Research Design

Fundamental qualitative description was the research design selected to guide sampling, data collection and analysis procedures [26]. This type of qualitative approach is used to provide a comprehensive summary of facts and events, using the 'everyday' language of the participants, and is commonly used by researchers who require answers to questions about specific events or phenomena [26]. Permission to conduct this research was granted through the Research Ethics Boards of the Faculty of Health Sciences, McMaster University, Hamilton, Ontario, Canada and Makerere University in Kampala, Uganda.

### Setting

This research was conducted in Kampala, Uganda and participants were recruited from two non-governmental organizations (NGOs) that provide instrumental and social support to HIV/AIDS orphans. Both NGOs were located in semi-urban districts surrounding Kampala and provided service to urban and semi-urban populations within these districts. Both NGOs deliver emotional support to children and youth through informal and sporadic talk-therapy sessions usually scheduled at the directive of the youth or on drop-in basis. One NGO provided primary medical assistance and monitoring of anti-retroviral (ARV) medication administered to the HIV-positive youth.

The composition of the research team reflected a collaborative effort between researchers from McMaster University and Makerere University with leadership and research direction provided by the African researchers (JS, RK) and methodological and content expertise from the Canadian researchers (SH, SJ). Diversity of team members was intentional to ensure culturally sensitive recruitment and data collection procedures, to ensure the development of qualitative questions that would have meaning to Ugandan youths, and promote a culturally relevant interpretation of the data in the analysis stage.

### Participants

Using convenience sampling, a purposeful sample of 13 orphaned Ugandan youth currently attending one of the two NGOs was recruited to participate in the study. Study eligibility criteria consisted of: 1) death of one or both parents to HIV/AIDS; 2) age between 12 and 18 years; 3) affiliation with an NGO providing support to orphans; 4) able to communicate in either English or Luganda; and 4) no suspected or diagnosed learning disability.

### Data Collection

Data were collected by individual in-depth semi-structured interviews from January to June 2006. This qualitative data collection strategy best facilitates the discussion and in-depth exploration of participants' experiences and perceptions of key events [28]. This type of interview includes a series of structured, pre-determined open-ended questions developed to explore the phenomenon under study and that provide flexibility for the researcher to further probe and co-create meaning about new concepts that emerge within the context of the interview. An interview guide was developed [6] and adapted as data collection progressed. The interview guide and interview probes were translated into Luganda and then back-translated to ensure accuracy. Demographic data were collected using a short, written questionnaire administered by the research assistant and each participant was also asked to complete a family genogram to illustrate family relationships, organization and structure. The qualitative interviews were scheduled at a time, date and location that was convenient for the youth. Both interviewers were clinically trained to deal with adolescent psychological and emotional distress. Opportunities for post-interview debriefing and clinical follow-up were offered to all youth given the sensitive nature of the material being discussed in the interviews. Transportation costs to the interview site were covered and a small meal was provided to each participant. All youth participants who were approached agreed to participate in the study.

Each interview lasted between 90 to 120 minutes, permission to record each interview was obtained and they were conducted in the local language of Luganda. Field notes were also completed at the end of each interview by the researcher to describe the context of the interview and to highlight emerging key themes. Seven of the interviews were conducted by J.S. and the remaining six interviews were conducted by Ugandan psychiatrist, R.K. Both interviewers received training in qualitative interviewing techniques to ensure dependability. In order to minimize researcher effects on the participants such as inducing social behaviours that would not have typically occurred [29], the Canadian researchers were not present for any of the key informant interviews. All field notes, analytic memos, the research log and a case summary of each interview were combined to create the study audit trail.

### Data Analysis

As common in qualitative research, participant recruitment, data collection and analysis occurred simultaneously. The computer software package, QSR NVivo 2.0 (QSR, 2002a) was used to manage and code the data. Given the exploratory nature of this study, all transcripts, memos and case summary data were analyzed using qualitative content analysis [29,30]. All transcripts were initially read in their entirety and then a process of line-by-line coding was conducted. Initial codes were developed using concepts from the interview guide. Subsequently, second-level coding was conducted by collapsing initial codes into broader categories, identifying category properties and establishing the relationships between categories [30]. Researcher assumptions were documented prior to the initiation of data collection and referred to during the data analysis as a means of strengthening objectivity or confirmability [31]. To promote the dependability of the emerging findings, multiple transcripts were independently coded by three members of the research team (SH, RK, & SJ).

## Results

Thirteen Ugandan youths supported by local NGOs, including five males and eight females participated in this study. The mean age of orphaned youth was 15 years. Six of the youth were HIV-positive, six were HIV-negative and the status of one youth was unknown. Of the orphaned youth, seven of them had lost both parents to HIV/AIDS, five were paternal orphans and one was a maternal orphan. In total, seven participants came from polygamous homes. All of the youth had reached some level of high school education however none of them had completed high school. Six of the youth were not attending school at the time of the study. The length of time being orphaned varied from two months to 13 years. The majority of the youth were from the Baganda tribe which is the predominant tribe in the south-central region of Uganda. All of the youth were from urban or semi-urban homes. Orphaned youth spoke extensively about the loss of their childhood, their exposure to extended family conflict and their experiences of being socially stigmatized.

### The Experience of an HIV/AIDS Orphan

#### The End of Childhood

"If my parent was alive, I would be playing instead of digging."

All participants, irrespective of HIV/AIDS status, described several types of losses during the time of parental illness and death. However, the most poignant losses were the actual death of a parent, lost educational opportunities and the loss of familial property and land. Participants also indicated that their experiences of childhood were markedly changed through prolonged absences from school, increased manual work responsibilities, increased financial responsibilities, and the requirement to care for siblings. During the interviews, the sharing of these experiences was often followed by long periods of silence or tears. With respect to educational losses, access to education was clearly associated with hopes for obtaining gainful employment to provide basic needs for self and one's family. Consequently, the loss of educational opportunities was particularly salient for many of the youth, as illustrated by this comment:

OK, you may be there when you don't have someone helping you, someone taking care of you. You don't have food. You don't have someone providing you with this and that. And even when you are not going to school, you may be there all the time worried because you don't have someone to pay your school fees and you can't see your future. This too brings problems.

#### Orphanhood Begins with Parental Illness

The youth unanimously talked about their experience as orphans beginning with the illnesses of their parents as opposed to the deaths of their parents. Many of the orphaned youth described watching their parents' physical suffering as something that they would never be able to forget. This time of parental illness was also marked by extended periods of school absenteeism to be the primary caregiver for the sick parent. As one 16 year old female explained,

I have seen a lot right from the time when my father was still alive.... when he was sick. We had to drop out of school and sit home to nurse him. All of the money was spent on treating him. We didn't have our own house, and the landlord where we were renting sent us away. We even lacked shelter.

Families struggling to pay for additional medical expenses to care for sick parents often did not have adequate resources to pay for food, shelter or the youths' school fees. One of the female participants explained,

There was even one term that we sat home without attending school. There was nothing to do. No school fees. We didn't have food. Nothing to eat. We were just crying. And I said, 'O God! You've left me here to sit without going to school.' What could I do?

In additional to the tangible losses, the period of parental illness was also characterized by difficult emotional losses for the youth. For example, one 14-year-old youth described her parent as a confidant who was irreplaceable. Youth also talked about an overwhelming uncertainty and sadness that permeated this time. Others identified having fears about what the future would hold for them following the deaths of their parents.

#### Conflict with the Clan

Eleven of the youth described the time around the death of the parent to HIV/AIDS as being marked by conflict with extended family and relatives around the issue of property ownership. As one participant described:

When he [father] was still in hospital, the relatives from my mother's side were trying to fight for our property, wanting to take everything because even mother was sick. They were praying hard that father never recovers so that they [could] take the house-hold property.

Other property left by the dead parent for the remaining children was perceived as an opportunity by other clan members to take advantage of the orphan's vulnerable position and to claim these limited resources. One youth illustrated this scenario with the following description:

But there were some who were happy because he [father] was going to die and leave this property. The moment he died, we started seeing some clan members that we didn't even know. Some of them had never come home to visit us and we didn't even know them. Moreover, you saw that they wanted to sell most of the property. Eventually we [the siblings] realized that they were preparing to sell it to some rich man.

Following the death of their parent(s), 12 of the 13 youth moved in with extended family. Most of these living situations were described as very difficult circumstances marked by discrimination, unequal treatment compared to other children in the home, stigmatization and again, experiencing not having basic needs being met. This experience was similar between HIV-positive and HIV-negative youth although the HIV-positive youth described more severe discrimination and, at times, disclosed experiencing overt physical abuse. One 15-year-old HIV-positive female youth disclosed:

[My new caregiver] started abusing me all the time saying, 'That girl is a grown up and she is even a prostitute.' I wanted to go back to school but she refused because she wanted me to stay home and look after her children. And one day she threatened to send me away and threw out my property. And sometimes she beats me. When she beats me, she uses a lot of force.

However, not all HIV/AIDS orphaned youth had exclusively negative experiences with extended relatives. Two of the paternally orphaned youth described having a family member that they trusted and who remained an advocate for them despite very difficult living situations.

#### "Okulangira"

One type of stigmatization that was repeatedly described by the orphaned youth was referred to in the local language of Luganda as "okulangira." This is a verbal exchange where one person is reminded in a derogatory manner about his/her inferior societal position. "Okulangira" in this context was described as when new caregivers announced to the community that the youth's parent had died of HIV/AIDS. It was perceived that the caregivers did this to invoke gratitude from the child for undertaking the task of caring for an HIV/AIDS orphan. Another type of stigma included being perceived as HIV- positive by peers, school mates or community members because the parent had died of HIV/AIDS. In cases where the individual was actually HIV-positive, youth stated that relatives or caregivers would often disclose this confidential information to the community without their expressed permission. These reported experiences of stigma led to relationship conflicts and frequent isolation for the orphaned youth. As one participant shared:

And now all the clan people, the relatives, are isolating us. They are running away from us. Everyone has abandoned us. Because those relatives do not even want to come to our home anymore. They are saying, 'Those children are moving corpses. We are about to bury them.' And this caused a lot of pain to me.

## Discussion

This qualitative study was conducted with a homogenous, purposeful sample of youth receiving services specifically for HIV/AIDS orphans through NGO organizations in Uganda. While the sample size of this study is small, data saturation occurred by focussing interview questions on a narrow phenomenon. Data credibility was achieved through triangulation of multiple types of data and the use of multiple researchers, including two professionals with expertise in mental health, HIV/AIDS orphans and cultural knowledge of Uganda. Data dependability was achieved by involving the full research team in the analytic process and in the double-coding of multiple transcripts to ensure that the most relevant concepts were identified. The findings of this study will only be transferable and of relevance to individuals working with adolescent HIV/AIDS orphans in Sub-Saharan Africa who regularly access support from NGOs.

However, the sample size of the current study necessitates caution when extrapolating findings to broad populations of youth orphaned by HIV/AIDS who are involved in NGOs. Additionally, the potential influence of NGOs providing informal talk-therapy on the orphan narrative is an important consideration. While many youth may have benefited from the talk-therapy provided at the NGO, the possibility remains that the recollection of orphaning experiences has been influenced. Specifically, the experience of receiving empathic and caring responses from NGO staff in response to telling personal orphan narratives could affect how subsequent narratives are told. The possibility of receiving monetary support through an NGO also has the possibility to effect their narratives in a way that inadvertently rewards stories of hardship or sensationalism. However, services provided through NGOs in support for HIV/AIDS orphans are one of the few available resources to this group of vulnerable children and youth given the significant limitations for domestic funds or available health infrastructure in Uganda [32].

The loss of a parent as a child or an adolescent is significant in any culture; however the onset of parental illness described in the current study marked the beginning of a series of unwanted impending changes for many of the study participants. The effects of parental HIV illness on children and youth has been studied primarily in a western context with results uniformly showing that children and youth experience significant levels of psychological distress during this period [33].

Through structural equation modelling, Rotherham-Borus and colleagues [34] examined the impact of parental death secondary to HIV/AIDS on New York adolescents and also studied the effectiveness of an intervention model to assist with adolescent coping in this context. In general, emotional distress and problem behaviours were highly correlated with time in youth who had lost a parent to HIV/AIDS. Girls were described as experiencing more emotional difficulties but fewer behavioural problems compared to males at baseline and at two years. Male youth had more behavioural difficulties at baseline. Interventions to assist with coping were found to be effective in reducing numbers of sexual partners and behavioural difficulties compared to controls. Another study [35] looked at six-year outcomes for American youth who had lost parents to HIV/AIDS. While there were significant differences between bereaved and non-bereaved youth on outcomes related to emotional distress, contact with the criminal justice system and negative life events surrounding the time of parental death, these differences disappeared at the time of one year follow-up. Rotheram-Borus and colleagues [36] also conducted a randomized control trial in New York where teens of HIV-positive parents were provided an intervention consisting of opportunities for dialogue between the HIV-positive caregiver and their teens as compared to a control situation which included care as per usual. Outcomes at a six-year follow-up suggested that those youth who participated in the intervention group did significantly better on outcomes such as income, use of welfare, education and interpersonal relationships as compared to controls.

While findings from western literature suggest that youth of HIV-positive parents experience psychological difficulty at the time of parental illness but have better long-term outcomes with psychological support, it is difficult to generalize this data to the current study for several reasons. Most notable are differences for African youth where death is processed differently within the cultural context such that they are often unaware or uncertain of the realities of the parental HIV/AIDS illness and death. In a recent qualitative study by Withell [37] where HIV/AIDS affected Ugandan youth talked about their psychological needs living with a dying parent, most youth revealed that realities with respect to nature of their parents' illness and impending death were disguised or concealed. They described feeling unprepared for what lay ahead as well as feeling isolated. Wood, Chase and Aggleton [3] also reported descriptive narratives from Zimbabwean orphaned teens that had lost parents to HIV/AIDS. Many of the youth in this study described difficult experiences associated with being care givers to their ill parents, watching their parents deteriorate and then witnessing the eventual death. However, many of the youth also described the experience of not being told what the cause of illness and death was as being equally stressful. The traditional approach to death was described as hiding the reality of death from youth and keeping them from funerals. Youth in the study described being rewarded for remaining silent during bereavement as a sign of effective coping. These youth indicated that they would have preferred honest conversations in order to prepare them for what was ahead. Many of the adults also found themselves unprepared to converse with the teens about grief and impending parental death. The suggestions of these African youth raise important questions about the utility and tension of challenging cultural norms related to bereavement, particularly when the direction of change being suggested may be perceived as reflection of Western culture. However, the notion that support is needed at the time immediately surrounding parental death is a common theme emerging from both African and Western literature.

Ugandan youth in the current study also described experiencing psychological distress with the perceived loss of childhood and the subsequent need to prematurely assume adult roles. This may be referred to as a process of "parentification." This phenomenon was reported throughout the time of parental illness as well as into the period of time living with extended relatives. Although little African data is available in this regard, work done by Murphy and colleagues [38] in Los Angeles attempted to determine the impact of parentification on adolescent's development of autonomy, which is considered a normal and healthy psychological progression during the teen years. Samples consisted of uninfected teens of 108 HIV-positive mothers. Findings indicated that youth who took on care-taking roles showed better development of autonomy in early and middle adolescence suggesting that parentification may not negatively affect development of autonomy. These findings, as they relate to the current study, may suggest that Ugandan youth who participate in caring for their sick parent may not result in negative developmental experiences. However, this responsibility coupled with additional stressful experiences such as lost educational opportunities, poverty and potential mental health disorders may increase the risk for poorer developmental outcomes with respect to the attainment of a healthy sense of autonomy.

Several of the youth alluded to being exploited in their new home situations following the death of a parent(s). Examples were cited describing unequal workload distribution within the home or orphaned youth being treated more harshly compared to other children. Specifically, orphaned youth described having to leave school after moving in with the extended family whereas other children in the home continued their education. While the psychological distress that was reported by youth after the loss of a parent(s) can not be disputed, the notion that orphaned children and youth have been frankly exploited as a ubiquitous phenomenon has been challenged recently in the literature. Work done by South African researchers [39] attempted to determine potential differences between school-aged HIV/AIDS orphans and same aged children who lived in the same house on several measures including education, nutritional status, labour, and psychosocial status. Their findings suggested that the "Cinderella myth" which contends that HIV/AIDS orphans are exploited in their adopted homes was not supported. There were no statistically significant differences between children on most of the variables except for education. Paternal orphans tended to be behind in schooling compared to same age peers. While this data is specific to children, it may be that the Ugandan youth in the current study actually do experience exploitation which has not yet been empirically validated.

In this small sample, the reported loss of educational opportunities within the context of parental illness or death was a salient and concerning theme. The long-term impact of not completing education or some type of skills training is arguably of grave importance in a low-income country such as Uganda where employability and income generation is often associated with basic survival. Some African studies have attempted to better understand the school status of HIV/AIDS orphaned children and youth. A large scale retrospective study conducted in Malawi by Floyd and colleagues [40] compared school participation between children of HIV-positive versus HIV-negative parents. Results indicated that HIV/AIDS status did not affect primary school participation for children 15 years and younger. However, children of HIV-positive parents were less likely to complete secondary school compared to children of HIV-negative parents. Timaeus and Boler [41] compared school progress of maternal and paternal orphans, ages eight to 20 years residing in a South African province known to have high HIV/AIDS mortality rates. Results indicated that living with a well-educated mother was beneficial to a child however there was no evidence to suggest that being a maternal orphan or living apart from one's mother adversely affected one's school progress. Conversely, being a paternal orphan or living outside the father's household resulted in slower school progress.

Data in the current study revealed limited educational opportunities for HIV/AIDS orphans, which has generally been supported by previous research findings. A sub-analysis regarding gender and missed educational opportunities could not be conducted in the current study given the limited sample size. All of the orphans had lost educational opportunities, however the magnitude of these losses was not explored. This information may lead to important findings such as identifying those most at risk of school removal as well as subsequent long-term developmental trajectories associated with school losses. An important consideration that has not been previously studied is the potential experience of social poverty for youth who are forced to leave school or for those who experience stigma related to their HIV/AIDS orphan status in this setting. The social cohesion within the educational setting may be an important buffer for orphaned youth, particularly for ones who experience "okulangira" - social exchanges where orphaned youth were stigmatized by being reminded of their inferior social status in the extended family or community. The potential additive effects of social erosion within the traditional African surrogate family system due to multiples stressors and strains associated with the HIV/AIDS crisis may make orphaned youth twice or thrice vulnerable with respect to social losses. In a study by Cluver and Orkin [42], South African youth orphaned by HIV/AIDS were found to have much higher chances of psychological distress when poverty and stigma were present. In this study, stigma included comments and responses from the extended family, peers and community. However, a previous study conducted in South Africa [43] found that reduction of HIV/AIDS related stigma could potentially reduce adverse psychological outcomes for AIDS orphans. These findings suggest that more research is needed with respect to determining where social losses are experienced most saliently for youth orphaned by HIV and more importantly, to determine in what settings to focus on establishing networks of resilience.

## Conclusions

The experience of becoming an HIV/AIDS orphan in the current study is a dynamic process marked by several difficulties and challenges for Ugandan youth. The experience of orphanhood begins with parental illness, not death. The implications of impending parental death to HIV/AIDS herald the onset of several struggles including poverty, lost educational opportunities, living with extended family systems marked by difficulty, potential exploitation within their homes and culturally specific forms of stigma related to their HIV/AIDS orphan status making these youth twice and thrice vulnerable. While there is much more work to do in attempting to better understand this heterogeneous population and phenomenon, it is clear that a moral and ethical imperative exists to better understand how to provide meaningful support and care for a vulnerable population during and after the tragedy of losing a parent(s) to HIV/AIDS.

## Competing interests

The authors declare that they have no competing interests.

## Authors' contributions

SH, SJ and RK participated in study design. RK and JS participated in data collection. SH, SJ and RK conducted data analysis. SH and SJ drafted the manuscript. SH, SJ, JS and RK reviewed and edited drafts of the manuscript.
